# ApoE mimetic peptide decreases Aβ production *in vitro *and *in vivo*

**DOI:** 10.1186/1750-1326-5-16

**Published:** 2010-04-20

**Authors:** S Sakura Minami, Antoinette Cordova, John R Cirrito, Joseph A Tesoriero, Lenard W Babus, Gary C Davis, Sivanesan Dakshanamurthy, R Scott Turner, Daniel TS Pak, G William Rebeck, Mikell Paige, Hyang-Sook Hoe

**Affiliations:** 1 Department of Neuroscience, Georgetown University, 3970 Reservoir Rd. NW, Washington, DC 20057, USA; 2 Drug Discovery and Drug Therapeutics, Georgetown University, 3970 Reservoir Rd. NW, Washington, DC 20057, USA; 3 Department of Neurology, Georgetown University, 4000 Reservoir Rd. NW, Washington, DC 20057, USA; 4 Department of Pharmacology, Georgetown University Medical Center, 3900 Reservoir Rd. NW, Washington, DC 20057, USA; 5 Department of Neurology, Washington University, St. Louis, MO 63110, USA

## Abstract

**Background:**

Apolipoprotein E (apoE) is postulated to affect brain Aβ levels through multiple mechanisms--by altering amyloid precursor protein (APP) processing, Aβ degradation, and Aβ clearance. We previously showed that an apoE-derived peptide containing a double repeat of the receptor-binding region was similarly effective in increasing APP processing *in vivo*. Here, we further examined whether peptides containing tandem repeats of the apoE receptor-binding region (amino acids 141-149) affected APP trafficking, APP processing, and Aβ production.

**Results:**

We found that peptides containing a double or triple tandem repeat of the apoE receptor-binding region, LRKLRKRLL, increased cell surface APP and decreased Aβ levels in PS1-overexpressing PS70 cells and in primary neurons. This effect was potentiated by a sequential increase in the number of apoE receptor-binding domain repeats (trimer > dimer > monomer). We previously showed that the apoE dimer increased APP CTF *in vivo*; to determine whether the dimer also affected secreted APP or Aβ levels, we performed a single hippocampal injection of the apoE dimer in wild-type mice and analyzed its effect on APP processing. We found increased sAPPα and decreased Aβ levels at 24 hrs after treatment, suggesting that the apoE dimer may increase α-secretase cleavage.

**Conclusions:**

These data suggest that small peptides consisting of tandem repeats of the apoE receptor-binding region are sufficient to alter APP trafficking and processing. The potency of these peptides increased with increasing repeats of the receptor binding domain of apoE. In addition, *in vivo *administration of the apoE peptide (dimer) increased sAPPα and decreased Aβ levels in wild-type mice. Overall, these findings contribute to our understanding of the effects of apoE on APP processing and Aβ production both *in vitro *and *in vivo*.

## Background

Alzheimer's disease (AD) is an age-related neurodegenerative disease characterized by the progressive loss of synapses and neurons and by the formation of amyloid plaques and neurofibrillary tangles [1]. Amyloid plaques are composed predominantly of the Aβ peptide, a 40 or 42 amino acid cleavage product of the amyloid precursor protein (APP). APP is a synaptic, transmembrane protein that undergoes extracellular cleavage by one of two proteases, α- or β-secretase, which results in the formation of large N-terminal extracellular fragments of secreted APP (sAPP) and smaller, membrane-bound C-terminal fragments (CTF). If the initial cleavage event occurs via β-secretase, then cleavage of the CTF by γ-secretase in the intramembrane region results in the formation of Aβ. Mutations in APP and in a component of the γ-secretase complex (presenilin) cause familial forms of AD. APP can be preferentially cleaved by α- or β-secretase depending on its localization within the cell. The majority of α-secretase activity is thought to occur on the cell surface [2], whereas β-secretase cleavage and Aβ production is thought to occur following endocytosis of APP in the endosomal pathway [3]. Understanding APP function, trafficking, and processing in neurons may provide valuable information in generating interventions against AD pathogenesis and its accompanying memory loss.

ApoE is the major genetic risk factor for AD (reviewed in Roses, 2006 [4]) and is known to directly bind Aβ and co-localize with cerebral amyloid deposits in AD patients [5-8]. However, the mechanism by which apoE and its receptors affect the risk for AD remains unknown. Some possible effects of apoE include altering APP processing [9], facilitating internalization and degradation of Aβ [10-13], improving clearance of Aβ into the periphery [14], and altering neuronal toxicity of Aβ [15]. However, some studies show detrimental effects of apoE where apoE facilitates Aβ oligomerization [16,17]. Some of the contrasting evidence may be attributed to the different isoforms of apoE: human apoE2 reduces brain Aβ burden in transgenic APP mice, while human apoE4 increases brain Aβ burden [18]. These isoform specific effects were also seen in APP transgenic mice expressing human apoE, where Aβ deposition was greatest in apoE4 APP transgenic mice compared to apoE3 and apoE2 [19,20]. Recombinant human apoE at physiological levels (100 nM) has been reported to decrease Aβ production in CHO-APP751, HEK293, and primary neuron cells [21]. However, other studies show that lipid deficient apoE4 in APP-overexpressing rat neuroblastoma B103 cells increased Aβ production compared to lipid deficient apoE3 [22], and apoE binding to apoEr2 promoted APP endocytosis, increasing Aβ production [23]. Thus, there is no consensus yet about how apoE affects APP processing.

We and others [24-26] have used a derivative of apoE, a small peptide containing a tandem repeat of the receptor binding domain, to show the effects of apoE on neuronal signaling and APP processing. We found that apoE-derived peptide treatment increased ERK and decreased JNK activation in primary neurons [27]. In addition, the apoE-derived peptide significantly reduced inflammation in several animal models of disease [28,29], which may occur through the apoE peptide-induced decrease in c-Jun N-terminal kinase-mediated microglial activation [30]. Furthermore, we chronically administered the apoE-derived peptide via osmotic pump and observed a consistent effect on apoE signaling, as well as on APP processing, *in vivo *[25]. These data implicate a role for the apoE-derived peptide, regardless of isoform, in various signaling processes in neurons and glia, and these signaling processes may be related to our observed effects on altered APP processing.

In the present study, we investigated the importance of the tandem repeat in the apoE-derived peptide, and the ability of this peptide to affect APP trafficking, APP processing, and Aβ levels. We first demonstrate that a tandem repeat in the apoE-derived peptide is necessary to effect cell surface APP levels. We then show that apoE-derived peptides decrease secreted Aβ in PS1-overexpressed PS70 cells and in primary neurons. Again, the effect of the monomer was minimal in reducing secreted Aβ levels in both systems, whereas the dimer and trimer show a clear dose response. The potency of the apoE-derived peptides follows the order: trimer > dimer > monomer, which correlates with the stability of their alpha helical secondary structure as determined computationally. The effect of the dimer on APP processing was further evaluated *in vivo*, where a single hippocampal injection of the apoE-derived dimer peptide resulted in a significant increase in sAPPα and a significant decrease in soluble Aβ1-40 levels in wild-type mice, suggesting that the apoE dimer may promote α-secretase cleavage. Our data demonstrate a clear effect of the apoE-derived dimer peptide on APP trafficking, processing, and Aβ levels both *in vitro *and *in vivo*.

## Results

### Characterization of apoE peptides

ApoE-derived peptides have been found in several studies, including our own, to be involved in reducing inflammation and in increasing APP processing [25,26,30]. This peptide sequence was derived from the receptor-binding domain of apoE (LRKLRKRLL). In our previously published studies, we used an apoE-derived peptide that consisted of a tandem repeat of this sequence, labeled as the dimer in this study (Table 1). To test the importance of the tandem repeat of the apoE-derived mimetic peptide in affecting APP processing and Aβ production, we also synthesized the single LRKLRKRLL sequence denoted as the monomer, and the triple tandem repeat, (LRKLRKRLL)_3_, denoted as the trimer (Table 1).

**Table 1:** Amino acid sequences of the apoE-derived peptides used.

*Peptide*	*Sequence*
Monomer	LRKLRKRLL
Dimer	LRKLRKRLLLRKLRKRLL
Trimer	LRKLRKRLLLRKLRKRLLLRKLRKRLL

Based on the structure of the apoE receptor binding domain, we hypothesized that these peptides could exist as alpha helices with varying degrees of stability, which may result in differences in binding efficacy. Therefore, a computational study was employed to determine the relative stability of the three apoE-derived peptides as alpha helices. As shown in Table 2, the alpha helical structure of the trimer is considerably more stable than the dimer by approximately 2.4 kcal/mol, and the alpha helical structure of the dimer is more stable than the monomer by approximately 2.0 kcal/mol.

**Table 2:** ΔE values for monomer, dimer, and trimer apoE-derived peptides.

*Peptide*	Δ*E (kcal/mol)*
Monomer	6.75
Dimer	8.77
Trimer	11.21

Energy change of each structure was computed by molecular simulations as described.

The propensity of the LRKLRKRLL motif to form alpha helices was further investigated by a search through the Dihedral Angle and Secondary Structure Database of Short Amino Acid Fragments (DASSD). [REFERENCE: Dayalan S, Gooneratne ND, Bevinakoppa S, Schroder H: **Dihedral angle and secondary structure database of short amino acid fragments ***Bioinformation *2006, 1:78-80] The DASSD database stores the dihedral angles and the secondary structure details of short amino acid fragments derived from 5,227 non-redundant protein structures with less than 2 Å resolution. A search of the LRK motif revealed that 222 out 311 structures (71%) in the DASSD database contained the LRK motif in alpha helices according to STRIDE classification. Similarly, a search of the RLL motif revealed that 300 out of 476 structures (63%) contained this sequence in an alpha helix. A representative sample of unique structures that contain these motifs in alpha helical structures are given by the following PDB codes: 1EWK, 1YKE, 2CWY, 1WUD, 1CII, 2GHJ, 3KS9, 2QR4, and 1TTY. This supports our hypothesis that the LRK and RLL motifs in our apoE-derived peptides have propensities toward forming alpha helical structures.

### ApoE dimer is not toxic to PS70 cells or primary neurons

**Figure 1:**
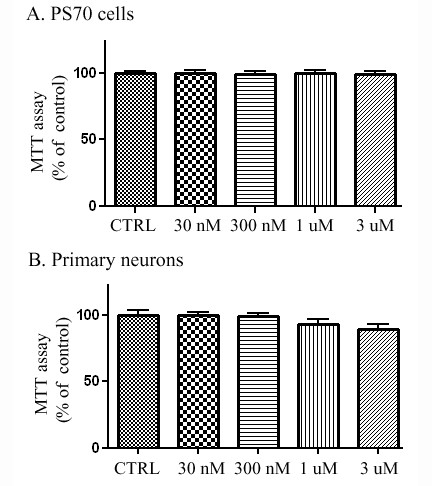
**ApoE-derived peptide does not affect cell viability in PS70 or primary neuronal cells**. **A**. PS70 cells overexpressing PS1 were treated with apoE dimer at various concentrations for 24 h and cell viability was assayed (n = 6). ApoE dimer treatment did not result in cell toxicity at any of the indicated concentrations. **B**. Primary neurons were treated with apoE dimer at various concentrations for 24 h and cell viability was assayed (n = 6). ApoE dimer treatment did not result in cell toxicity at any of the indicated concentrations.

In order to first test the potential toxicity of our compounds, we treated PS70 cells (a PS1-overexpressing cell line which produces high levels of Aβ) with PBS control or increasing doses of the apoE-derived peptide dimer (30 nM, 300 nM, 1 uM, 3 uM) for 24 h and conducted an MTT assay to evaluate for cell viability. We found that there was no toxicity of the peptide compared to control at any concentration tested (Fig. 1A). We also tested for toxicity of this compound in primary neuronal cells. Again, we did not observe any toxicity at any of the concentrations tested compared to control (Fig. 1B).

### ApoE dimer and trimer promote surface levels of APP in COS7 cells

**Figure 2:**
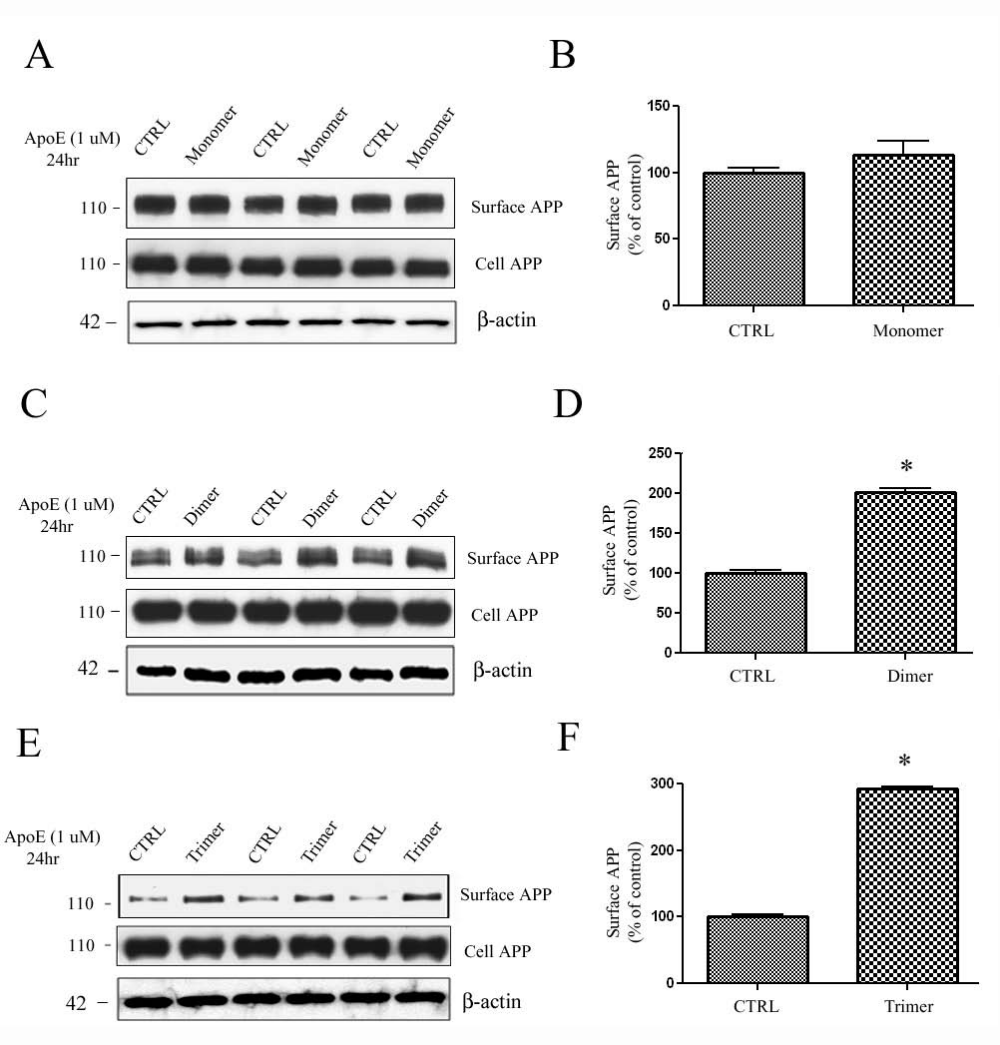
**ApoE-derived peptide regulates APP trafficking in COS7 cells**. COS7 cells were transiently transfected with APP and treated with control or apoE monomer (n = 3, **A**), apoE dimer (n = 3, **C**), or apoE trimer (n = 3, **E**) for 24 h. Total APP and β-actin levels in cell lysates were measured for loading control (lower panels). Quantification of biotin-labeled cell surface APP in COS7 cells showed a 12% (**B**), 98% (**D**), and 197% (**F**) increase in surface APP by the apoE monomer (NS: no significant), dimer, and trimer, respectively (n = 3, *p < 0.01). Levels of β-actin in the cell lysate were consistent across conditions (bottom panels).

Trafficking of APP to the cell surface may be important for effects on APP processing, as specific secretases are known to be distinctly localized within the cell. β- and γ-secretases are predominantly present intracellularly in endosomes, and α-secretase is active at the cell surface [31,32]. To examine the effects of apoE peptides on cell surface levels of APP, we transfected COS7 cells with APP and treated cells with control, 1 μM monomer, 1 μM dimer, or 1 μM trimer for 24 h. Cell surface proteins were biotin labeled, isolated with avidin beads, and immunoblotted for APP. Monomer treatment caused a non-significant 12% increase in cell surface levels of APP (Fig. 2A &2B). The dimer and the trimer treatments significantly increased cell surface levels of APP by 98% and 197%, respectively (Fig. 2C-F). Levels of cell β-actin were consistent across conditions, serving as a loading control (Fig. 2A-C, bottom panels). These data suggest that the tandem repeat of the receptor-binding region of apoE is necessary for our apoE-derived peptides to affect APP trafficking.

### ApoE-derived peptides increase cell surface APP in primary cortical and hippocampal neurons

**Figure 3:**
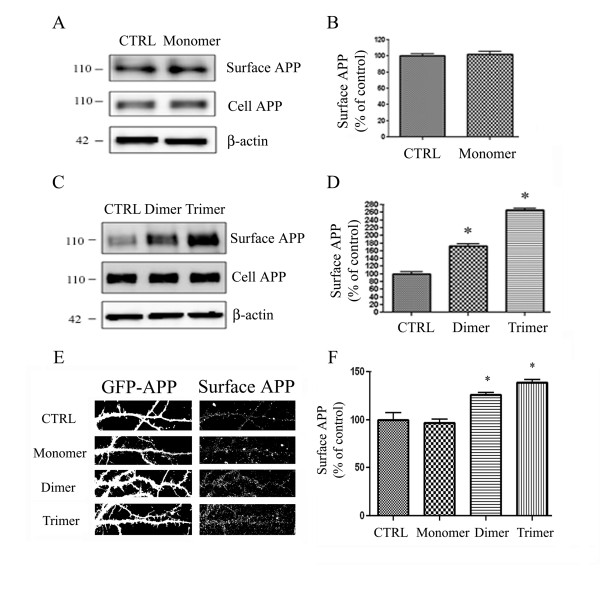
**ApoE-derived peptide affects APP trafficking in primary cortical and hippocampal neuronal cells**. **A**. Cultured cortical neuronal cells were treated with control (PBS) or apoE monomer (n = 3, 1 μM for 24 hr). Cell surface proteins were biotinylated, isolated with avidin-conjugated beads, and immunoblotted with 22C11 antibody against rodent APP. Total APP and β-actin in cell lysates were measured in the bottom panel. **B**. Quantification of biotin-labeled cell surface APP in primary cortical neurons showed no significant change by apoE monomer compared to control. **C**. Cultured cortical neuronal cells were treated with control (PBS), apoE dimer, or apoE trimer (n = 3, 1 μM for 24 hr). Total APP and β-actin in cell lysates were measured in the bottom panel. **D**. Quantification of biotin-labeled cell surface APP showed a 72% (p < 0.01, n = 3) and 166% (p < 0.001, n = 3) increase in surface APP by the apoE dimer and trimer, respectively. Error bars indicate SEM. **E**. Cultured hippocampal neurons were transfected at DIV12 with GFP conjugated APP. At DIV 14 cells were treated with PBS control or the apoE monomer (n = 10, 1 μM), dimer (n = 10, 1 μM), or trimer (n = 10, 1 μM) for 24 h, and surface APP was measured by live cell staining. **F**. Quantification of surface APP intensity in (**A**) showed a 21% and 43% increase by the dimer and trimer, respectively (n = 10, *p < 0.05, *p < 0.01).

To test whether the apoE mimetic peptides regulate endogenous APP trafficking in primary cortical neuronal cells, we treated cells with control (PBS) or 1 μM apoE monomer for 24 hrs. Cell surface proteins were biotinylated, isolated with avidin beads, and immunoblotted for APP with antibody 22C11. ApoE monomer did not alter cell surface levels of APP (Fig. 3A-B).

We also examined the effect of apoE dimer and trimer on surface APP levels in cultured cortical neurons by treating with 1 μM apoE dimer or trimer for 24 hrs and found increased surface levels of endogenous APP after 24 hrs of apoE dimer (by 72%, n = 3, p < 0.01) and trimer (by 166%, n = 3, p < 0.001) treatment (Fig. 3C-D). These results are consistent with those obtained in COS7 cells (Fig. 2), where we observed an effect of apoE dimer and trimer, but not monomer, on increased cell surface APP levels.

To further test whether the apoE mimetic peptides regulate APP trafficking in primary hippocampal neurons, we transfected hippocampal neurons with N-terminal GFP-tagged APP and treated cells with control or 1 μM of the apoE monomer, dimer, or trimer for 24 h. Transfection of cells with APP allowed us to measure surface APP by live cell immunostaining. The dimer and trimer treatments significantly increased cell surface levels of APP (Fig. 3E, third and fourth panels). Quantification of these data showed significant 21% and 43% increases in surface APP by the dimer and trimer in primary hippocampal neuronal cells, respectively (n = 10, *p < 0.05, *p < 0.01, Fig. 3F). These data are consistent with our cell surface biotinylation results, and support a role for apoE dimer and trimer peptides in altering APP trafficking.

### ApoE-derived peptides significantly decrease Aβ levels in PS70 cells

**Figure 4:**
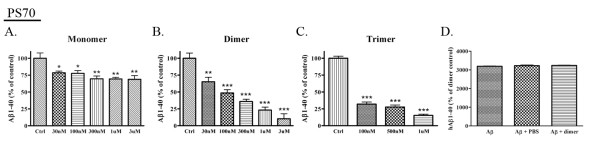
**ApoE-derived peptides significantly decrease Aβ1-40 levels in PS70 cells**. PS70 cells were treated with the apoE monomer (n = 6, **A**), dimer (n = 6, **B**), or trimer (n = 6, **C**) for 24 h at various concentrations. Aβ1-40 levels in the conditioned media were determined by ELISA (n = 6). The apoE monomer decreased Aβ1-40 by 20% at 30 nM and 100 nM (p < 0.05) and by 30% at 300 nM, 1 μM, and 3 μM (p < 0.01). The apoE dimer decreased Aβ1-40 by 35% at 30 nM (p < 0.01), 51% at 100 nM, 64% at 300 nM, 77% at 1 μM, and 90% at 3 μM (p < 0.001 for all). The apoE trimer decreased Aβ1-40 by 68% at 100 nM, 72% at 500 nM, and 85% at 1 μM (p < 0.001 for all). **D**. Human Aβ1-40 was measured from samples containing the apoE dimer peptide alone, synthetic human Aβ40 alone, synthetic human Aβ40 with PBS, or synthetic human Aβ40 with apoE dimer peptide. Aβ1-40 values were normalized to background with apoE dimer peptide alone, and were not found to be significantly different in the presence of PBS or apoE dimer peptide.

The above data suggest that the apoE mimetics may decrease β-cleavage of APP by changing APP trafficking to favor α-cleavage. To directly test whether the apoE-derived peptide analogs could affect Aβ production, we treated a human PS1-overexpressing cell line (PS70) with the apoE mimetic peptides at various concentrations (30 nM to 3 μM) for 24 h and measured Aβ1-40 levels from the conditioned media by ELISA. We found that treatment with the apoE monomer resulted in small but significant decreases in Aβ1-40 compared to control (20% decreases at 30 nM and 100 nM (p < 0.05); 30% decreases at 300 nM, 1 μM, and 3 μM (p < 0.01) (n = 7 for all concentrations except 3 μM, n = 5) (Fig. 4A)). The dimer and trimer resulted in larger significant dose-dependent decreases in Aβ1-40 (dimer: 35% decrease at 30 nM (p < 0.01); 51% decrease at 100 nM; 64% decrease at 300 nM; 77% decrease at 1 μM; 90% decrease at 3 μM (each p < 0.001); trimer: 68% decrease at 100 nM; 72% decrease at 500 nM; 85% decrease at 1 μM (each p < 0.001) (n = 6 for all dimer concentrations and for the control trimer; n = 4 for all concentrations of the trimer) (Fig. 4B-C)). Again, we observed a consistent effect where the monomer was least effective and the trimer was most effective in reducing Aβ1-40 levels. These data suggest that increasing the number of tandem repeats of the receptor binding domain increases the potency of the apoE mimetic peptides in lowering Aβ. We performed a control experiment to demonstrate that the apoE peptide did not interfere with the ELISA assay, and found that measurement of human Aβ1-40 was not different in the presence of PBS or apoE dimer peptide (Fig. 4D).

### ApoE-derived peptides significantly decrease Aβ levels in primary neuronal cells

**Figure 5:**
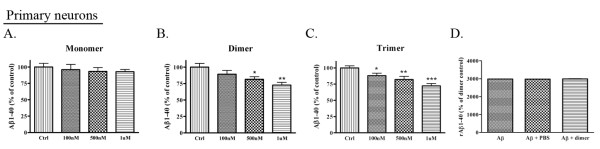
**ApoE-derived peptides significantly decrease Aβ1-40 levels in primary cortical neuronal cells**. Primary cortical neurons were treated with apoE monomer (n = 6, **A**), dimer (n = 6, **B**), or trimer (n = 6, **C**) for 24 h, and Aβ1-40 levels in the conditioned media were determined by ELISA (n = 6). Treatment of primary neurons with apoE dimer decreased Aβ1-40 by 19% at 500 nM (p < 0.05) and 27% at 1 μM (p < 0.01). Treatment with apoE trimer decreased Aβ1-40 by 12% at 100 nM (p < 0.05), 18% at 500 nM (p < 0.05), and 28% at 1 μM (p < 0.001). **D**. Rodent Aβ1-40 was measured from samples containing the apoE dimer peptide alone, synthetic rodent Aβ40 alone, synthetic rodent Aβ40 with PBS, or synthetic rodent Aβ40 with apoE dimer peptide. Aβ1-40 values were normalized to background with apoE dimer peptide alone, and were not found to be significantly different in the presence of PBS or apoE dimer peptide.

In order to determine whether the apoE peptide could affect Aβ production in a more relevant system, we treated primary neuronal cells (DIV 14) with various concentrations of the apoE peptides (monomer, dimer, or trimer) and measured rodent Aβ1-40 by ELISA after 24 h. Treatment with the apoE monomer did not result in significant decreases in Aβ1-40 (Fig. 5A) (n = 5), but treatment with the apoE dimer and trimer resulted in significant dose-dependent decreases in Aβ1-40 (dimer: 19% decrease at 500 nM (p < 0.05); 27% decrease at 1 μM (p < 0.01) (n = 5) (Fig. 5B); trimer: 12% decrease at 100 nM (p < 0.05); 18% decrease at 500 nM (p < 0.05); 28% decrease at 1 μM (p < 0.001) (n = 5) (Fig. 5C)). As with our previous experiment, we performed a control experiment to demonstrate that the apoE peptide did not interfere with the ELISA assay, and found that measurement of rodent Aβ1-40 was not different in the presence of PBS or apoE dimer peptide (Fig. 5D).

### ApoE-derived peptide increases secreted APPα and decreases Aβ production *in vivo*

**Figure 6:**
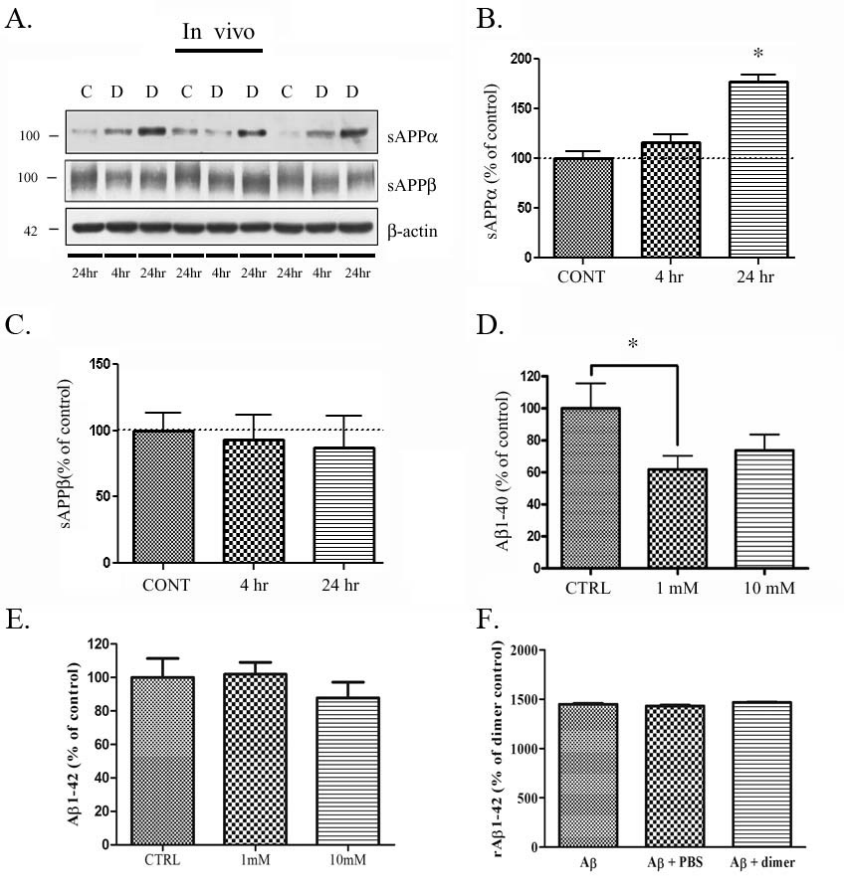
**ApoE-derived peptide affects APP processing and Aβ production *in vivo***. **A**. sAPPα was measured from wild-type mice injected with vehicle control (C) for 4 h (n = 6), apoE dimer (D) for 4 h (n = 6) or apoE dimer (D) for 24 h (n = 6). Wild-type mice showed an increase in sAPPα after 24 h and a slight increase in sAPPα after 4 h compared with control treated mice. **B**. Quantification of experiments demonstrated that injection with the apoE dimer significantly increased sAPPα by 77% after a single injection (*p < 0.01). **C**. Quantification of experiments demonstrated that injection with the apoE dimer did not alter sAPPβ. **D**. Aβ1-40 was measured from wild-type mice injected with PBS for control (n = 9), or apoE dimer at 1 mM (n = 9) or 10 mM (n = 8) for 24 h. ApoE dimer-treated mice showed a 40% decrease in Aβ1-40 at 1 mM after 24 h and a slight 26% decrease in Aβ1-40 at 10 mM after 24 h compared with control-treated mice. **E**. Aβ1-42 was measured from the same animals and was not significantly different in either 1 mM or 10 mM injected animals compared to control. **F**. Rodent Aβ1-42 was measured from samples containing the apoE dimer peptide alone, synthetic rodent Aβ42 alone, synthetic rodent Aβ42 with PBS, or synthetic rodent Aβ42 with apoE dimer peptide. Aβ1-42 values were normalized to background with apoE dimer peptide alone, and were not found to be significantly different in the presence of PBS or apoE dimer peptide.

We have thus far demonstrated that the apoE dimer regulates APP trafficking and Aβ production *in vitro*; to test whether the dimer affected endogenous APP proteolysis *in vivo*, we injected PBS control or a 10 mM solution of the dimer (in a 2 μL volume) into the hippocampus of wild-type mice. After 4 h or 24 h, we dissected the tissue surrounding the injection site from each brain and measured endogenous rodent sAPPα and sAPPβ by western blot. We found that sAPPα was not altered at 4 h, but was significantly increased by 77% at 24 h by the dimer compared to controls (Fig. 6A &6B, upper panel, n = 6). We did not find significant differences in sAPPβ compared to controls (Fig. 6A &6C, second panel, n = 6). β-actin loading controls were consistent across samples (Fig. 6A, bottom panel).

We did not observe a significant difference in Aβ40 levels in mice treated with 10 mM apoE dimer at 4 hrs or 24 hrs (data not shown). To test whether a lower, more physiologically relevant, concentration of the dimer affected endogenous Aβ production *in vivo*, we injected 1 mM or 10 mM solutions of the dimer (in a 2 μL volume) into the hippocampus of wild-type mice. We analyzed endogenous rodent Aβ1-40 by ELISA (Fig. 6D). We found that Aβ1-40 was significantly decreased by 40% with the 1 mM apoE dimer injection after 24 h treatment (n = 9) compared to controls (PBS, n = 9) (Fig. 6D). We found a similar, but not statistically significant, decrease in Aβ1-40 with the 10 mM apoE dimer injection after 24 h (n = 8). We also tested whether endogenous rodent Aβ1-42 levels were altered with treatment. We did not observe any significant difference in Aβ1-42 levels following apoE peptide treatment at 1 mM or 10 mM (Fig. 6E). To test whether the dimer interfered with rodent Aβ1-42 ELISA detection, we assayed for rodent Aβ1-42 in the presence of PBS or apoE dimer peptide and found no significant difference compared to Aβ1-42 alone (Fig. 6F). These data suggest that a single 1 mM injection of apoE dimer lowers endogenous Aβ1-40 production *in vivo*.

## Discussion

In these studies, we tested whether single (monomer), double (dimer), or triple (trimer) repeats of the receptor-binding domain of apoE had an effect on Aβ levels *in vitro *and *in vivo *and whether this effect was accompanied by changes in APP trafficking or processing. We showed that the apoE dimer and trimer increased cell surface APP levels *in vitro *(Fig. 2 &3). We also observed a dose-dependent decrease in secreted Aβ levels from PS70 cells and primary neurons treated with either dimer or trimer (Fig. 4 &5). Finally, we found an *in vivo *effect of apoE dimer treatment, where we observed increased sAPPα and decreased Aβ at 24 hours following a single hippocampal injection of the apoE dimer (Fig. 6). These data support the idea that the apoE receptor-binding domain, especially in either double or triple tandem repeat form, significantly increases cell surface APP and secretion of sAPPα, and decreases Aβ levels.

Previous studies have implicated a role for apoE, and the apoE mimetic peptide, in modulating APP processing by increasing APP CTFs both *in vitro *and *in vivo *[21,25]. We hypothesized that regulation of APP trafficking could mediate this effect, and found that the apoE dimer and trimer increased cell surface levels of APP *in vitro *(Fig. 2 &3). This increase in cell surface APP could lead to reduced availability of APP to cleavage by β- and γ-secretases, which are predominantly present in early endosomes and may promote α-secretase cleavage, resulting in the previously observed increase in APP CTFs and decrease in Aβ production.

Full-length apoE has been shown to decrease secreted Aβ levels *in vitro *[21], and here, we show that the apoE monomer, dimer, and trimer were sufficient to lower Aβ levels in human PS1-overexpressing PS70 cells (Fig. 4). Although the apoE monomer efficiently reduced Aβ levels, it was much less effective than the apoE dimer or trimer. The apoE dimer and trimer induced a significant dose-dependent decrease in Aβ, and the apoE trimer further reduced secreted Aβ levels compared to the apoE dimer. We observed a similar, albeit less dramatic, decrease in rodent Aβ from primary hippocampal neurons (Fig. 5). This decrease was most apparent in response to the apoE trimer, less so in response to the apoE dimer, and not significant in response to the apoE monomer. We hypothesize that we were able to observe a much greater effect in PS70 cells due to the abundant levels of APP and Aβ in this system. Our investigation of endogenous Aβ production in primary neurons demonstrates an important effect of apoE peptide on Aβ production in the absence of overexpression. In addition, the data from both PS70 cells and primary neurons are consistent in supporting the idea that additional repeats of the receptor-binding domain significantly increase the effect of the apoE-derived peptides on lowering secreted Aβ levels, as well as on increasing APP trafficking.

Thus, we hypothesize that increasing the number of residues confers greater stability of the alpha helical structure, which results in a more rigid conformation and consequently reduced overall energy for binding. Computational analyses revealed that the stability of the alpha helix of the apoE-derived peptides are on the order of trimer > dimer > monomer, where the trimer is 2.4 kcal/mol more stable than the dimer and the dimer is 2.0 kcal/mol more stable than the monomer. Further structural analyses including NMR spectroscopy, x-ray crystallography, and circular dichroism spectroscopy should be employed in future studies to determine the three-dimensional structure of these peptides and test the hypothesis that increasing the number of receptor binding domain repeats increases the alpha helical nature of the peptide. Alternatively, we hypothesize that multiple receptor-binding domains may increase the ability of the peptide to bind multiple ligands, thus increasing its effect on APP trafficking and processing. However, these possibilities are not mutually exclusive, as increased stability of the alpha helix may contribute to the peptide binding multiple ligands.

Importantly, when we tested whether the apoE-derived peptide affected APP processing and Aβ levels *in vivo*, we found that 24 hours of treatment with the apoE dimer resulted in a significant increase in sAPPα levels (Fig. 6A). sAPPβ levels were unchanged (Fig. 6C); however, we observed a significant decrease in soluble Aβ1-40, but not Aβ1-42, levels following treatment with the apoE dimer (Fig. 6D). We hypothesize that we were not able to detect significant differences in Aβ1-42 due to the low levels of endogenous Aβ1-42 produced in the wild-type mouse. It will be interesting for future studies to determine whether apoE peptides can alter Aβ1-42 in APP overexpressing mice. It is important to note that at least two mechanisms contribute to the total measured levels of soluble Aβ--one involving APP processing and Aβ production, and the other involving Aβ degradation and clearance [12,14]. Here, we show that the apoE-derived peptide dimer affects α-secretase cleavage of APP as well as Aβ levels *in vivo*. However, the observed decrease in Aβ could be due to either or both apoE-mediated alterations in APP processing and Aβ clearance. Full-length apoE has been shown to effectively internalize and degrade Aβ through neurons and microglia [11,33]. Thus, our data suggest that the major mechanism underlying apoE-mediated Aβ lowering may involve alterations to APP trafficking and α-secretase processing.

## Conclusions

In summary, we provide data supporting a role for an apoE mimetic peptide in increasing trafficking of APP to the cell surface, increasing α-secretase cleavage of APP, and decreasing Aβ levels *in vitro *and *in vivo*, which is facilitated by the presence of multiple receptor-binding tandem repeats. These data implicate new roles for apoE in APP trafficking as well as in APP processing, and provide further evidence that apoE, regardless of isoform, decreases Aβ levels in wild-type mice. In addition, the present study provides new insight regarding the structure-based action of apoE on APP trafficking and processing and Aβ production.

## Methods

### Chemicals

The apoE-derived peptides consist of the monomer (LRKLRKRLL), the dimer (LRKLRKRLLLRKLRKRLL), and the trimer (LRKLRKRLLLRKLRKRLLLRKLRKRLL). The monomer represents the receptor-binding region of apoE (amino acids 141 through 149), and the dimer and trimer represent double and triple tandem repeats of the monomer, respectively. The peptides were synthesized by standard solid phase peptide synthesis (SPPS) with acetylation of the N-terminus and amidation at the C-terminus. Synthesis of the peptides was contracted through Biomatik USA, LCC (Wilmington, DE). The dimer peptide has signaling properties comparable to full-length apoE (Tolar et al., 1999), and does not contain amino acids that differ between apoE isoforms. We used antibody 6E10 (Signet) for detecting cell surface APP and C1/6.1 (from Dr. Paul Matthews) for total APP in biotinylation experiments. We used an APP N-terminal antibody (Sigma) to detect surface APP in live cell surface staining experiments. Antibody clone 2B3 (IBL, Gunma, Japan) was used to detect sAPPα, a rabbit polyclonal sAPPβ antibody was used to detect wild-type sAPPβ (IBL), and monoclonal β-actin antibody (sigma) was used to confirm loading control.

### Computation Methods

The monomer, dimer and trimer structural models were based primarily on the x-ray structure of apoE (PDB:1GS9). The E values indicate the energy change of each structure after energy minimization. The apoE monomer, dimer and trimer were energy minimized using consistent valence force field (CFF91) with the default partial atomic charges available in Discover, Insight II (Accelrys Inc.). The cutoff for nonbonded interaction energies was set to ∞ (no cutoff). To avoid unrealistic movements of the peptide caused by computational artifacts, the structures were relaxed gradually. Each minimization was conducted in two steps, first using steepest descent minimization for 200 cycles and then using conjugate gradient minimization until the average gradient fell below 0.01 kcal/mol.

### Cell Lines and Culture Conditions

COS7 (monkey kidney) cells or PS70 (Chinese hamster ovarian (CHO) cells overexpressing wild-type human PS1 and producing high levels of Aβ [34]) cells were used for our experiments. Cells were maintained in Opti-MEM (**Invitrogen**) with 10% fetal bovine serum (**Invitrogen**) in a 5% CO_2 _incubator. COS7 cells were transiently transfected with 0.5-1 μg of plasmid in FuGENE 6 (Roche Applied Science) according to the manufacturer's protocol and cultured 24 h in Dulbecco's modified Eagle's medium containing 10% fetal bovine serum. After 24 h the cells were transferred to Opti-MEM serum-free media (**Invitrogen**) and treated with indicated apoE peptides. Cells were collected 24 h later for subsequent analyses.

### MTT Assay

MTT assay was performed in either PS70 cells or primary cortical neurons. 1 mg/ml MTT solution was prepared in PBS and 50 μl of this solution and 200 μl of DMEM without phenol red were added into each well. Cells were incubated for 4 hours at 37°C with 5% CO_2_. After 4 hours, the MTT solution was removed and replaced with 200 μl DMSO and 25 μl Sorenson's glycine buffer (glycine 0.1 M, NaCl 0.1 M, pH:10.5). The plate was further incubated for 5 min at room temperature, and the optical density (OD) was determined using a plate reader at a test wavelength of 570 nm and a reference wavelength of 630 nm.

### Cell surface biotinylation

COS7 cells were cultured 24 h in Opti-MEM containing 10% fetal bovine serum, then transferred to Opti-MEM serum-free media and treated with indicated apoE peptides. After 24 h, cells were washed twice with phosphate-buffered saline, and surface proteins were labeled with Sulfo-NHS-SS-Biotin, 500 μl at 500 μg/ml phosphate-buffered saline (Pierce) under gentle shaking at 4°C for 30 min. 50 μl of quenching solution (Pierce) was added to cells at 4°C, which were washed twice with Tris-buffered saline. Cells were lysed in 500 μl of lysis buffer, collected with a cell scraper, disrupted by sonication on ice, incubated for 30 min on ice, and clarified by centrifugation (10,000 × *g*, 2 min). To isolate biotin-labeled proteins, lysate was added to immobilized NeutroAvidin TM Gel (50 μl) and incubated for 1 h at room temperature. Gels were washed 5 times with wash buffer and incubated for 1 h with SDS-PAGE sample buffer including 50 mm dithiothreitol. Surface proteins were then analyzed by immunoblotting.

### Primary Neuronal Culture and Cell Surface Immunostaining

Primary hippocampal or cortical neurons from E18-19 Sprague-Dawley rats were cultured at 150 cells/mm^2 ^as described (17). Neurons were transfected with GFP conjugated APP at 10-12 days *in vitro *by calcium phosphate precipitation (4-5 μg of DNA/well). 2 days after transfection, cells were treated with apoE peptides for 24 h, and cell surface expression levels of APP were analyzed. Surface immunostaining was performed as described previously (18). Briefly, live neuron cultures were incubated with anti-APP N terminal antibody (10 μg/ml in conditioned medium) for 10 min to specifically label surface APP, then lightly fixed for 5 min in 4% paraformaldehyde (non-permeabilizing conditions). After fixation, the surface-remaining antibody labeled APP was measured with Alexa Fluor 555-linked α-rabbit secondary antibodies for 1 h. Images were collected using a Zeiss LSM510 confocal microscope (Carl Zeiss, Thornwood, NY). Confocal z-series image stacks encompassing entire neurons were analyzed using Metamorph software (Universal Imaging Corp., Downingtown, PA) (18).

### *In vivo* analysis of APP fragments

Mouse brains were homogenized in a 10× volume of 50 mM Tris-HCl buffer, pH 7.6, containing 250 mM sucrose and protease inhibitor cocktail (Sigma, St. Louis, MO). Soluble APP and Aβ were extracted in 0.4% DEA, as previously described (Nishitomi et al., 2006) Briefly, crude 10% brain homogenate was mixed with an equal volume of 0.4% DEA, sonicated, and ultracentrifuged for 1 hour at 100,000 × g. The supernatant was collected and neutralized with 10% 0.5 M Tris base, pH 6.8. The resulting DEA fraction was used for Western blot and ELISA analyses.

### Aβ ELISAs

Levels of endogenous full-length mouse Aβ1-40 from the media from primary neurons or wild-type brain DEA fractions were quantified using sandwich ELISA as previously described (Nishitomi et al., 2006). Briefly, a 96-well plate (Maxisorp) was coated with an anti-Aβ40 antibody, clone 1A10, overnight at 4°C. After blocking for 2 hrs, standards (synthetic mouse Aβ peptide 1-40) and samples were loaded and incubated overnight at 4°C. The plate was incubated with HRP-coupled detection antibody, 14F1, and visualized using a 3,3',5,5'-tetra methyl benzidine (TMB) substrate. Rodent Aβ1-42 levels were measured from wild-type brain DEA fractions by an ELISA kit purchased from Invitrogen (Carlsbad, CA).

Human Aβ1-40 levels were measured from the media of PS70 cells using sandwich ELISA as previously described (Horikoshi et al., 2004), using anti-Aβ40 antibody clone 1A10 as the capture antibody and antibody clone 82E1 as the detection antibody.

### Surgical procedures

For single brain injections, mice were anesthetized with ketamine/xylazine (Sigma) and placed in a stereotaxic apparatus (David Kopf Instruments, Tujunga, CA, USA). 2 μL vehicle (PBS) or 2 μL 10 mM apoE dimer peptide in PBS was injected into the dorsal hippocampus (AP = -1.0 mm, ML = +1.8 mm, DV = -2.2 mm) from the bregma according to Paxinos and Watson (1998). Solutions were continuously delivered over a duration of 4 minutes. After completion of each injection, the cannula was left in place for an additional 4 min in order to accomplish quantitative diffusion of the volume delivered. At the appropriate survival times, animals were sacrificed, and the hippocampus and surrounding cortex were dissected and collected.

### Statistical analysis

Experiments were repeated a minimum of three times unless otherwise noted. All data were analyzed using ANOVA with Graphpad Prism 5 software, using Tukey's multiple comparison test for post hoc analyses with significance determined as *p *< 0.05. Descriptive statistics are displayed as mean ± SEM.

## Competing interests

The authors declare that they have no competing interests.

## Authors' contributions

AC, SD, and MP performed structural analyses on the chemical compounds; SSM, JRC, HSH, JAT, LWB, and GCD conducted experiments. SSM, MP, and HSH prepared the manuscript, GWR, RST, and DTSP provided resources and valuable scientific insight, and MP and HSH supervised the overall project. All authors have read and approved the final manuscript.
